# Comparison of a novel free breathing SSFP sequence with standard breath held SSFP- a pilot study

**DOI:** 10.1186/1532-429X-17-S1-P395

**Published:** 2015-02-03

**Authors:** Idan Roifman, Rhonda Walcarius, Labonny Biswas, Kim A Connelly, Graham A Wright

**Affiliations:** 1 Medicine, Imaging Research Centre for Cardiac Interventions, Schulich Heart Centre, Sunnybrook Health Sciences Centre, University of Toronto, Toronto, ON, Canada; 2 Physical Sciences Platform, Sunnybrook Research Institute, Sunnybrook Health Sciences Centre, Toronto, ON, Canada

## Background

Despite advances in magnetic resonance imaging hardware and software, the acquisition of optimal cardiac magnetic resonance (CMR) images may be hampered by several factors. Most notably, scan length, the requirement for multiple breath holds and cardiac gating can all impact upon image quality. Real time cardiac imaging holds promise for reducing scan times via rapid acquisitions of images that are not reliant on breath holding or cardiac gating. Recently, a novel free breathing SSFP sequence was made available as part of a pre-release version of the HeartVista Cardiac Package, Version 1.0.0 (HeartVista, Inc.). Our goal was to compare the assessment of LV function between the HeartVista free breathing SSFP (FB-SSFP) sequence and standard FIESTA breath held SSFP.

## Methods

8 patients with chronic stable coronary artery disease were scanned on 1.5T GE Signa Excite scanner between Dec 2013 and June 2014. Each patient had both a traditional GE FIESTA SSFP sequence as well as the novel free breathing SSFP sequence. The American Heart Association/American College of Cardiology 17 segment model was employed. Each segment was independently graded by two CMR specialists as 1 (normal), 2 (hypokinetic) or 3 (akinetic). Subsequently, agreement between graded scores was analyzed via both simple and weighted kappa analysis. Further, simple and weighted kappa values were also used to assess inter-observer agreement of the FB-SSFP sequence between the two CMR specialists.

## Results

Overall image quality was graded as good in all 8 cases. A total of 136 segments were compared using the traditional FIESTA SSFP sequence with the free breathing SSFP sequence. Of these, 10 segments in the FB-SSFP were excluded due to artifact. No segments were excluded in the FIESTA group. Simple kappa values were 0.79 (95%CI 0.66-0.93) and weighted kappa values were 0.87 (95%CI 0.77-0.96) for agreement between the two sequences. Interobserver agreement for the FB-SSFP sequence was as follows: Simple Kappa = 0.78 (95%CI 0.61-0.94) and weighted kappa = 0.82 (95%CI 0.68-0.97).

## Conclusions

FB-SSFP showed very good agreement with standard techniques in the assessment of LV function. Furthermore, there was very good interobserver agreement for FB-SSFP. These preliminary results suggest that FB-SSFP sequence holds the potential to be utilized clinically in patients who are unable to breath-hold or have cardiac arrhythmia. Further work in larger patient cohorts is required in order to validate these results.

## Funding

Funding support for this project was provided from HeartVista Inc.

